# Cost‐effectiveness of virtual Mindfulness‐Based Cognitive Therapy on pregnancies at high risk of perinatal depression

**DOI:** 10.1002/pmf2.70305

**Published:** 2026-05-19

**Authors:** Riane Bradbury‐Huang, Lindsay Quinn, Ellen L. Tilden, Aaron B. Caughey

**Affiliations:** ^1^ Department of Psychology Barnard College of Columbia University New York USA; ^2^ Department of Obstetrics and Gynecology Oregon Health and Science University Portland USA

**Keywords:** cognitive behavioral therapy, cost‐effectiveness analysis, Mindfulness‐Based Cognitive Therapy, perinatal depression, prevention, preventive treatment

## Abstract

**Objective:**

Previous research has shown that preventive counseling for women at high risk of perinatal depression leads to a reduction in the development of perinatal depression and is cost‐effective. Barriers of cost, transit, time, and staffing limit accessibility of these services. We examined whether a theoretical, virtual, group, social worker (SW)‐led Mindfulness‐Based Cognitive Therapy (MBCT) program for patients at high risk of perinatal depression would be cost‐effective.

**Study design:**

We developed a decision‐analytic model using TreeAge to compare outcomes in pregnant patients at high risk of perinatal depression who received a preventive virtual group MBCT curriculum versus standard care. We used a theoretical cohort of 469,000 patients, representing the number of annual pregnancies to individuals with a history of depression in the United States. Outcomes included postpartum depression, maternal suicide, postpartum psychosis, preterm birth, neonatal death, and neurodevelopmental delay in addition to cost and quality‐adjusted life years (QALYs). The willingness‐to‐pay threshold was set to $100,000/QALY. Literature searches were used to identify model inputs, and sensitivity analyses were conducted to assess model robustness.

**Results:**

Within the theoretical cohort of 469,000 people, the virtual, group, SW‐led MBCT curriculum prevented 26,180 cases of postpartum depression, 1 maternal suicide, 2 cases of postpartum psychosis, 4044 preterm deliveries, 39 neonatal deaths, and 47 cases of neurodevelopmental delay. The MBCT strategy led to lower costs (saving $152,496,858) and better outcomes (15,052 gained QALYs), making it cost‐saving. Sensitivity analyses demonstrated that MBCT remained cost‐saving until treatment reached $800, and cost‐effective until $4014, both of which were significantly higher than the expected cost of MBCT delivery.

**Conclusion:**

We found that virtual SW‐led group MBCT led to better outcomes and lower costs for pregnant patients at high risk of depression. Given these findings, the benefits to high‐risk patients, and the shortage of mental health providers, such preventive mental health models should be examined for broader implementation.

## 1 INTRODUCTION

Perinatal depression is a large and growing issue, with prevalence rates estimated at 13.2% in the United States in 2018, and 14.0% internationally in 2022 [1, 2]. Risk factors include domestic abuse, low socioeconomic status, adolescent pregnancy, history of mental illness, perinatal substance use, and Black or Indigenous race [3]. First‐time mothers and patients with twin gestations also report higher rates of depressive symptoms postpartum [4, 5]. Perinatal depression is an emerging cause of maternal mortality and the leading cause of preventable maternal mortality, as shown by increases in rates of purposeful and accidental self‐harm [6]. Beyond the toll on the mother, perinatal depression has strong effects on the infant including increased preterm birth rates [7], low birth weight [8], and poor infant weight gain [9].

Perinatal mood and anxiety disorders collectively cost an estimated $5300 annually per mother–child dyad when left untreated [10], or up to $12,897 per dyad according to a more recent study from Montana [11]. Reducing the prevalence of perinatal mood and anxiety disorders through effective and accessible preventive care is key to avoiding these costs. Current maternity care systems often fail to reach this goal. While screening for perinatal mental health issues is universally recommended, only approximately 64% of pregnancies successfully receive this screening, with even lower rates being found when the patient is a woman of color or using Medicaid insurance [12, 13]. Additionally, close to 85% of those who screen positive are unable to access adequate evaluation or treatment [14]. The United States Preventive Services Task Force recommends cognitive behavioral therapy modalities as one of two counseling interventions for preventing perinatal depression [15]. Previous research has shown that referring at‐risk groups to preventive counseling rather than treating perinatal depression after it develops is cost‐effective [16]. This approach is associated with reduced chronic depression, preterm delivery, and sudden infant death syndrome (SIDS) rates. Mindfulness‐Based Cognitive Therapy (MBCT) is a powerful tool to address and prevent perinatal depression as well as reduce rates of adverse birth events. This modality incorporates cognitive‐behavioral therapy techniques with mindfulness and has been shown to decrease perinatal depression rates for patients with a history of depression by up to 74% compared to treatment as usual [17].

However, despite its proven efficacy, MBCT can be difficult for patients to access due to factors such as geographic barriers, time commitment, and insurance coverage [18]. One way to reduce these issues is offering MBCT virtually, in a group setting, and training social workers (SWs), rather than psychologists, to lead the sessions. Though currently underutilized, SWs can effectively deliver a wide range of perinatal mental health care interventions. Offering MBCT to patients in a group, virtual setting has been shown to be effective for preventing perinatal depression and the associated poor maternal and fetal outcomes [19]. During the pandemic, virtual healthcare became more commonplace and was shown to result in similar rates of patient satisfaction as in‐person care for prenatal appointments, as well as improved perception of access to care [20, 21]. Virtual care sessions are also associated with lower costs, wait times, and cancellations [22]. Group‐based prenatal care modalities are well‐established for offering high‐quality and satisfying care [23, 24].

The purpose of this study was to evaluate the outcomes and costs of virtual, group, SW‐led MBCT as an approach to prevent perinatal depression.

## 2 MATERIALS AND METHODS

**FIGURE 1:**
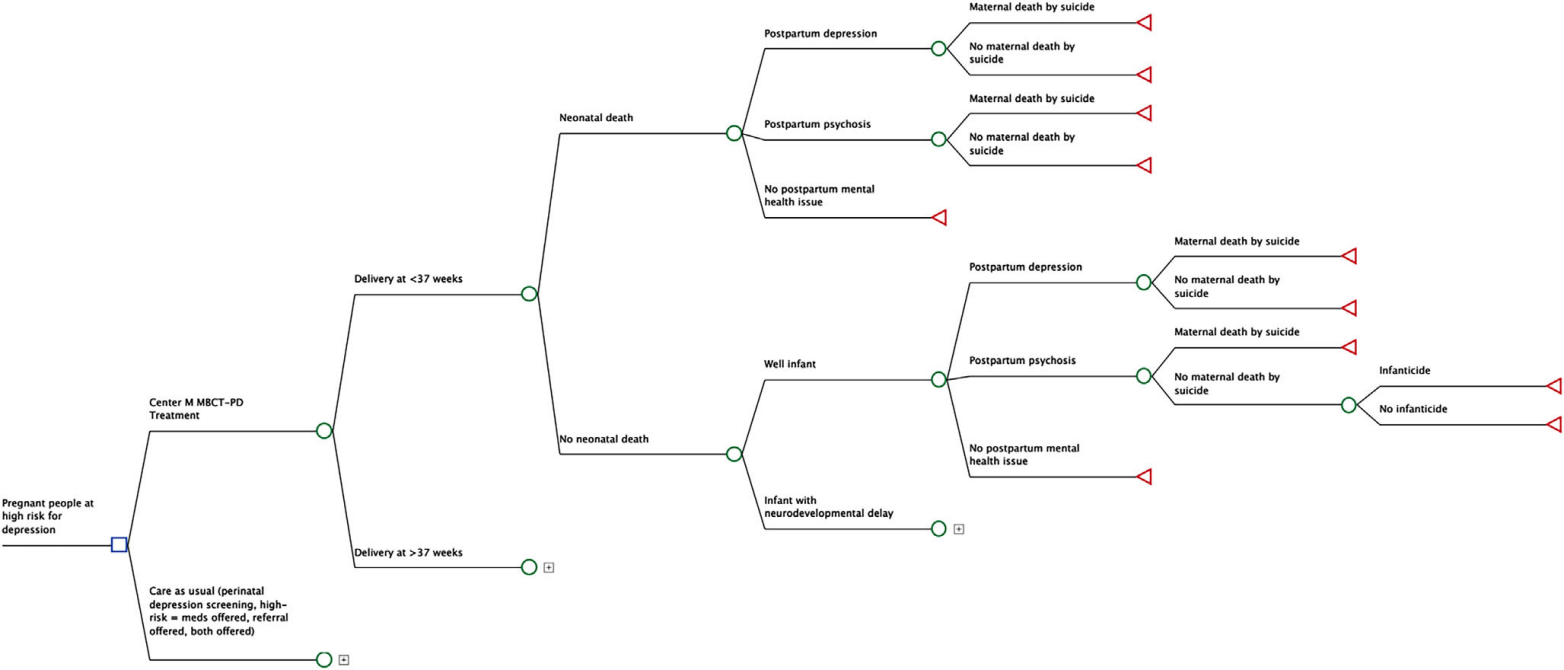
Cost‐effectiveness model. Plus signs indicate collapsed branches that match the displayed branches above. MBCT‐PD, Mindfulness‐Based Cognitive Therapy for perinatal depression.

TreeAge Pro software (2023 version; TreeAge Software, Inc) was used to create a decision‐analytic model comparing the outcomes and costs of virtual, group, SW‐led MBCT as opposed to treatment as usual. The initial decision node bifurcated into either receiving (1) MBCT treatment or (2) standard of care. All the following chance nodes represented either the presence or lack of each relevant health outcome—preterm birth, neonatal death, cases of perinatal depression, maternal suicide, cases of perinatal psychosis, infanticide, and cases of neurodevelopmental delay—with branches representing cases where a patient experiences all of the different possible combinations of the potential outcomes (Figure 1). Standard of care reflects existing care practices in which preventive mental health care is not routinely integrated or available in perinatal settings and was therefore defined as no preventive counseling and only treating those who developed perinatal depression [19, 25, 26]. This definition allows for inclusion of situations where depression screening is successfully implemented, but no follow‐up occurs despite patient scores recommending intervention [27]. The outcomes of this arm were abstracted from studies that did not include any form of counseling or therapy over the course of patients’ pregnancies. Given the relatively limited literature on MBCT and its effects on pregnancy outcomes due to its more recent adoption, efficacy rates of the primary pathway were sourced from studies on the impact of broader Mindfulness‐Based Interventions (MBIs) [28, 29, 30, 31, 32, 33], as well as the effect of standard cognitive behavioral therapy (CBT) to account for MBCT's unique nature that includes elements of both intervention methods. Additionally, a recent systematic review of randomized controlled trials from 2019 to 2021 studying the different impacts of MBIs and CBT found that participants showed no difference in anxiety and depression outcomes between the two approaches [34], thus further supporting the use of literature on similar methods to approximate MBCT impacts. Subsequent sensitivity analyses and simulations further reduced any slight discrepancies associated with intervention‐specific impacts on model outcomes by varying these rates within their ranges of uncertainty. Because a history of depression is a large risk factor for developing perinatal depression, the theoretical cohort was determined through the 2022 prevalence rate of depression in women, adjusted by the odds ratio of women with depression having children, and applied to the total number of live births in the United States in 2022 (3,664,292) [4, 35, 36]. This represented 12.8% of total pregnancies annually, or approximately 469,000 births per year. In actual obstetric practice, these patients could be identified through analysis of any past patient history of depression. Given that the USPSTF recommendation additionally identifies women with certain socioeconomic risk factors as an additional high‐risk population [15], this cohort likely represents an underestimation of the annual number of high‐risk pregnancies recommended for MBCT—though given this study's nature as a theoretical model, both the cohort and predicted health outcome cases are approximate estimates of the effect of preventive virtual, group MBCT.

The overall number of cases of each measured health outcome was compared between the two strategies. Additionally, the cost and effectiveness, measured through quality‐adjusted life years (QALYs), were determined for both strategies. QALYs were calculated by considering the utilities (quality‐of‐life preferences), from the maternal perspective, of each relevant outcome for its expected duration, with a discount factor of 3% every year. As is standard, the willingness‐to‐pay for every gained QALY was set at $100,000. This study was deemed exempt from Institutional Review Board approval as all inputs were taken from existing literature.

Baseline probability inputs were obtained from the literature (Table 1). The rate of suicide after receiving a diagnosis of perinatal depression was found from a study conducted between 2003 and 2007 that assessed outcomes during both pregnancy and within the first year postpartum [37]. A meta‐analysis on the existing literature regarding perinatal psychosis was used to find the rate of both maternal suicide and infanticide after receiving a diagnosis of perinatal psychosis [38]. The different rates of neurodevelopmental delay depending on term versus preterm delivery were found in a UK‐wide data analysis examining cerebral palsy rates [39]. A retrospective cohort study of births between 2003 and 2007 in California hospitals was used to determine rates of neonatal death depending on term or preterm delivery [40]. The effect of antepartum counseling on rates of preterm birth was determined through a randomized controlled trial showing that prenatal mindfulness programs lower perceived stress scores [28], followed by calculation of increased likelihood of preterm birth per point increase in perceived stress score adjusted by baseline preterm birth rates [35, 41]. The impact of neurodevelopmental delay on rates of perinatal depression was found in a randomized controlled trial on the association between cerebral palsy and postpartum depression [42]. A systematic review of risk factors for perinatal depression was used to identify the odds ratio of developing postpartum depression after a preterm delivery [43]. The odds ratio of developing perinatal psychosis after a preterm delivery was taken from a longitudinal cohort study of preterm deliveries in Quebec between 1989 and 2021 and was used to calculate the overall probability [44]. The overall probabilities of developing perinatal depression or perinatal psychosis after experiencing neonatal death were calculated using the odds ratio found in a longitudinal survey done in Michigan comparing 9‐month mental health outcomes for new mothers with mothers who experienced stillbirth or neonatal death within the first 28 days of life [45]. Finally, preventive counseling treatment was determined to lower rates of perinatal depression based on a systematic review of controlled trials involving primary care interventions to prevent perinatal depression during pregnancy or up to 1 year after childbirth [33]. It was assumed that preventive mental health care modalities would have no impact on the probability of developing perinatal psychosis based on the lack of existing literature. Similarly, adverse health outcomes were only assumed to increase perinatal psychosis risk if explicitly stated in the literature, as some complications were shown to uniquely affect perinatal depression risk but not perinatal psychosis risk—most notably, neurodevelopmental delay of the offspring [46].

**TABLE 1:** Probabilities used in the model.

Variable	Value	Range considered in sensitivity analysis	Citation
Prevalence of preterm birth	0.1049	0.01–0.3	[35]
Prevalence of neonatal death	0.0037	0.0001–0.009	[47]
Prevalence of maternal suicide	0.000011	0.000001–0.0001	[37]
Prevalence of postpartum depression	0.13	0.05–0.4	[1]
Prevalence of postpartum psychosis	0.0012	0.0001–0.009	[48]
Probability of suicide with perinatal depression	0.000056	0.00001–0.0005	[37]
Probability of suicide with perinatal psychosis	0.05	0.005–0.1	[38]
Probability of infanticide with perinatal psychosis	0.045	0.005–0.1	[38]
Probability of neurodevelopmental delay when preterm	0.013	0.005–0.1	[39]
Probability of neurodevelopmental delay when term	0.0011	0.0005–0.01	[39]
Probability of neonatal death when preterm	0.011	0.005–0.1	[40]
Probability of neonatal death when term	0.00091	0.0001–0.005	[40]
Probability of preterm birth with preventive counseling	0.096	0.1–0.15	[28, 35, 41]
Probability of perinatal depression when infant has neurodevelopmental delay	0.33	0.01–0.5	[42]
Probability of perinatal psychosis when infant has neurodevelopmental delay	0.0018	0.0005–0.01	[42]
Probability of perinatal depression after preterm delivery	0.196	0.05–0.4	[43]
Probability of perinatal psychosis after preterm delivery	0.0016	0.0009–0.009	[44]
Probability of perinatal depression after neonatal death	0.37	0.1–0.5	[45]
Probability of perinatal psychosis after neonatal death	0.0043	0.001–0.01	[45]
Probability of perinatal depression after preventive counseling	0.085	0.01–0.3	[33]
Odds ratio of having children with a history of depression	0.84	0.82–0.85	[36]

Costs were also obtained from the literature and adjusted to 2023 US dollars using the medical component of the Consumer Price Index (Table 2). The costs were determined from a societal perspective over a lifetime, or for as long as they were applicable. A cohort study of births in California between 1998 and 2000 was used to estimate the hospital costs associated with preterm versus term labor and delivery, as well as preterm neonatal death [49]. The cost of perinatal depression was taken from an estimate of the total direct costs associated with the diagnosis and treatment of major depression [50]. The cost of postpartum psychosis was represented by the average cost of a 5‐day inpatient psychiatric stay [51]. The cost of maternal death included the societal loss of expected wages between the mother's return to work and average retirement age; of note, there is inherent uncertainty in assuming these high costs given the wide variability of expected life span following birth, retirement age, and salary [52, 53, 54]. The cost of the MBCT treatments assumed that SWs were administering the virtual care in a group setting [19]. SW pay was calculated at $45/h, to encapsulate the upper range of SW salaries, the median of which was $29.49/h in 2024 [55]. A second version of the model was run only including costs faced by the health system rather than societal costs. This version did not include the associated costs with maternal death or lifetime treatment of a child with a neurodevelopmental disorder.

**TABLE 2:** Costs, utilities, and life expectancies used in the model.

Variable	Value	Range considered in sensitivity analysis	Citation
Cost of neonatal death, term	$119,066	$50,000–$300,000	[56]
Cost of neonatal death, preterm	$167,342	$50,000–$300,000	[49]
Cost of neurodevelopmental delay	$1,699,401	$500,000–$3,000,000	[57]
Cost of delivery, term	$3628	$1000–$5000	[49]
Cost of delivery, preterm	$33,313	$10,000–$70,000	[49]
Cost of perinatal depression	$6396	$1000–$10,000	[50]
Cost of postpartum psychosis	$7539	$1000–$10,000	[51]
Cost of maternal death	$2,051,094	$1,000,000–$5,000,000	[52, 53, 54]
Cost of preventive counseling sessions	$480	$10–$5000	[19]
Cost of prenatal care	$8853	$3000–$15,000	[58]
Utility of neurodevelopmental disorder	0.7333	0.6–0.8	[59]
Utility of neonatal death	0.76	0.6–0.8	[59]
Utility of preterm delivery	0.96	0.8–1	[60]
Utility of perinatal depression	0.7	0.5–0.9	[61]
Utility of postpartum psychosis	0.38	0.2–0.6	[61]
Utility of infanticide	0.5	0.2–0.8	See Section 2
Life expectancy of child with neurodevelopmental disorder	59.3 y	55–65	[62]
Life expectancy of healthy mother following pregnancy	54.8 y	50–60	[63]
Duration of perinatal depression	1.375 y	0.5–1.5	[61]
Duration of perinatal psychosis	1 y	0.5–1.5	[61]
Duration of infanticide impact	5 m	1 m–1 y	[61]

Utilities were abstracted from the existing literature. This study only utilized utilities from the maternal perspective given the study's focus on perinatal depression as a condition predominantly impacting mothers over infants (Table 2) [59, 60, 61, 62, 63]. Mounting evidence that perinatal depression negatively impacts fetal brain development and lifelong risk of mental health issues signals that examination of perinatal depression from the infant, family, or larger societal perspective may yield valuable insight in future analyses. Utilities were used to calculate QALYs and were applied for the appropriate duration of the mother's life. The utilities of preterm delivery and having a child with neurodevelopmental disorder were applied over the full life expectancy of the mother following pregnancy. The utility of infanticide was not found in the literature and was estimated with a utility that incorporated perinatal psychosis and neonatal death.

A method was cost‐saving if it was associated with lower costs and improved QALYs, and it was cost‐effective if there were higher costs, higher QALYs, and the incremental cost‐effectiveness ratio was less than $100,000/QALY. Univariate sensitivity analyses of the probabilities, costs, and utilities were performed to evaluate the impact of uncertainty within our model. A tornado diagram was used to find which inputs changed when the MBCT treatment was the cost‐effective strategy. Model inputs that were found to have a strong effect were further analyzed in a one‐way sensitivity analysis.

To examine the impact of uncertainty in the model simultaneously, a Monte Carlo analysis was run to simulate 10,000 trials that incorporated the distribution of each input in the model. Beta distributions were used for probability estimates, gamma distributions for cost estimates, and triangular distributions for the estimates of the duration of each outcome.

## 3 RESULTS

**FIGURE 2:**
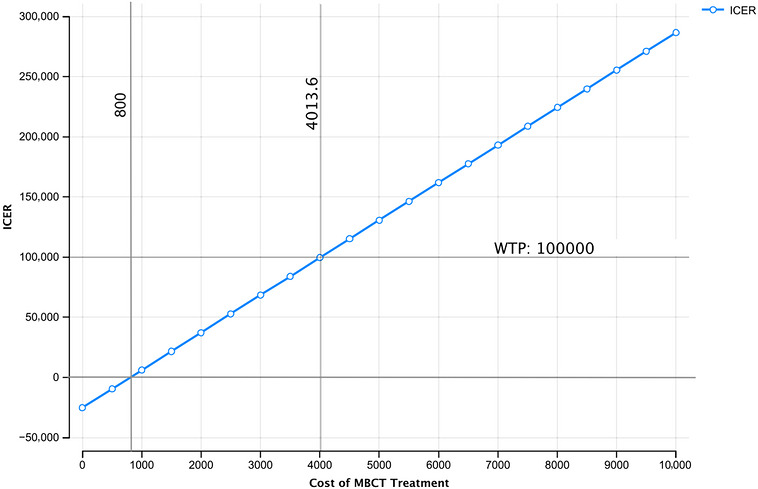
One‐way sensitivity analysis of cost of treatment and incremental cost‐effectiveness ratio (ICER). The baseline cost of Mindfulness‐Based Cognitive Therapy (MBCT) used in the model was $480. MBCT moves from cost‐saving cost‐effective when it becomes $800 and finally is no longer cost‐effective once it costs more than $4014. ICER, incremental cost‐effectiveness ratio; MBCT, Mindfulness‐Based Cognitive Therapy; WTP, willingness‐to‐pay.

Among the theoretical cohort of 469,000 high‐risk pregnancies in this model, preventive MBCT treatment resulted in 26,180 fewer cases of postpartum depression, 1 fewer maternal suicide, 2 fewer cases of postpartum psychosis, 4044 fewer preterm deliveries, 39 fewer neonatal deaths, and 47 fewer cases of neurodevelopmental delay (Table 3). Neonatal outcomes were included given their increased risk among patients who develop perinatal depression. These outcomes also served as contributors to overall costs and maternal QALYs. Additionally, the MBCT treatment approach was cost‐saving ($9,641,998,035 vs. $9,794,494,892). Given the improved outcomes and lower cost, the MBCT approach was a dominant strategy. A one‐way sensitivity analysis was run to examine the impact of increasing the cost of preventive MBCT; it remained cost‐saving until cost of treatment increased to $800 per dyad, and then cost‐effective until it reached $4014 per maternal/child dyad (Figure 2). When only hospital costs were considered, MBCT remained cost‐saving ($7,808,566,948 vs. $7,877,713,958).

**TABLE 3:** Outcomes prevented in a theoretical cohort of 469,000 pregnancies at high risk of postpartum depression assigned to either MBCT treatment or standard of care.

Outcome	Standard of care	MBCT treatment	Difference
Postpartum depression	66,565	40,385	−26,180
Maternal suicide	33	32	−1
Postpartum psychosis	587	585	−2
Infanticide	25	25	0
Preterm delivery	49,198	45,154	−4044
Neonatal death	905	866	−39
Neurodevelopmental delay	1088	1041	−47
Cost (USD)	$9,794,494,892	$9,641,998,035	−$152,496,858
Effectiveness (QALYs)	12,449,482	12,464,535	15,052

Abbreviations: MBCT, Mindfulness‐Based Cognitive Therapy; QALYs, quality‐adjusted life years; USD, US dollars.

**FIGURE 3:**
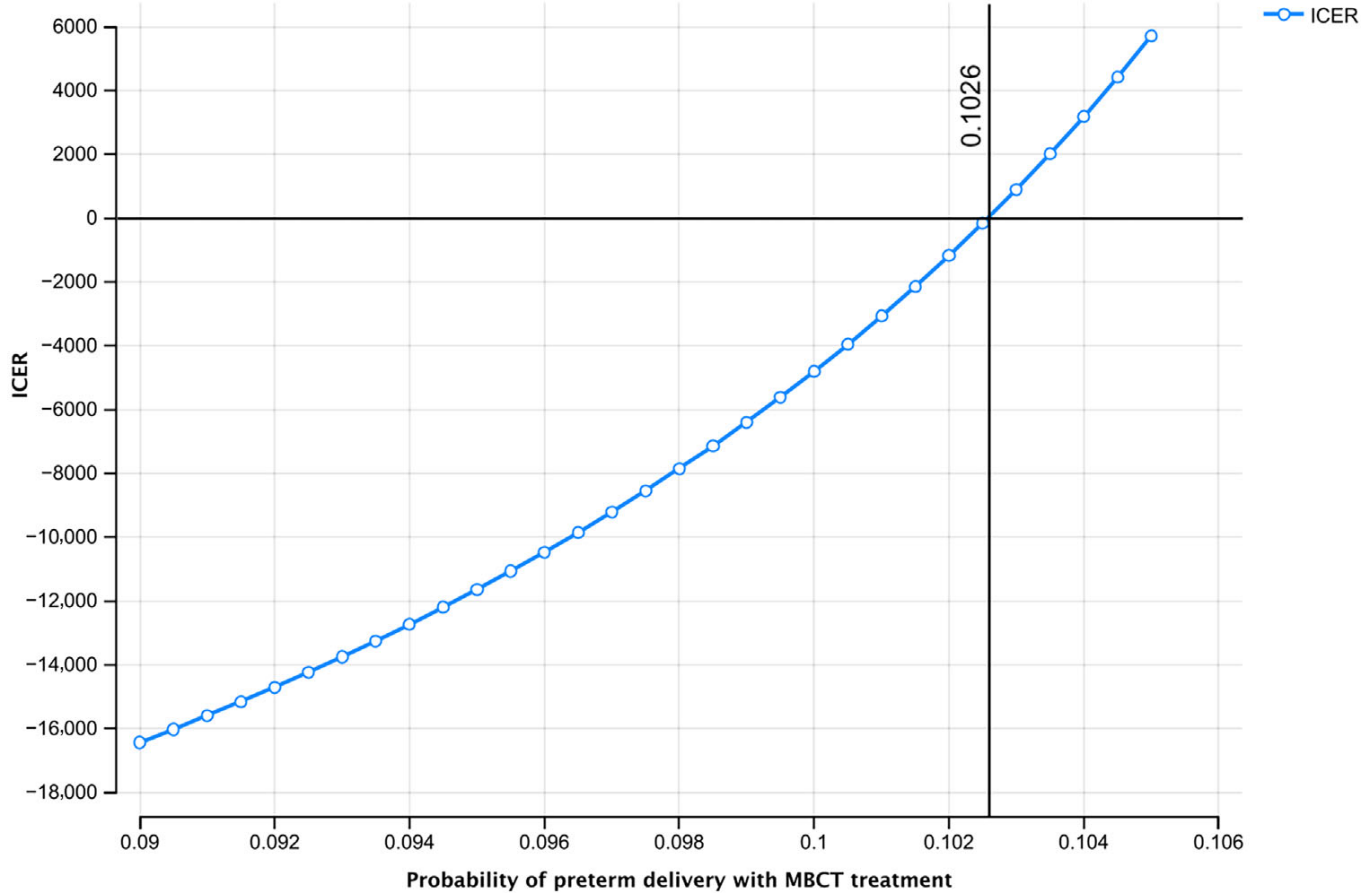
One‐way sensitivity analysis of the probability of preterm delivery with Mindfulness‐Based Cognitive Therapy (MBCT) preventive counseling. MBCT care remains cost‐saving until the chance of having a preterm delivery after treatment reaches 10.26%, at which point it becomes cost‐effective. This assumes that preterm delivery without treatment would stay at 10.5%. The baseline probability used in the model was 9.6%. ICER, incremental cost‐effectiveness ratio.

**FIGURE 4:**
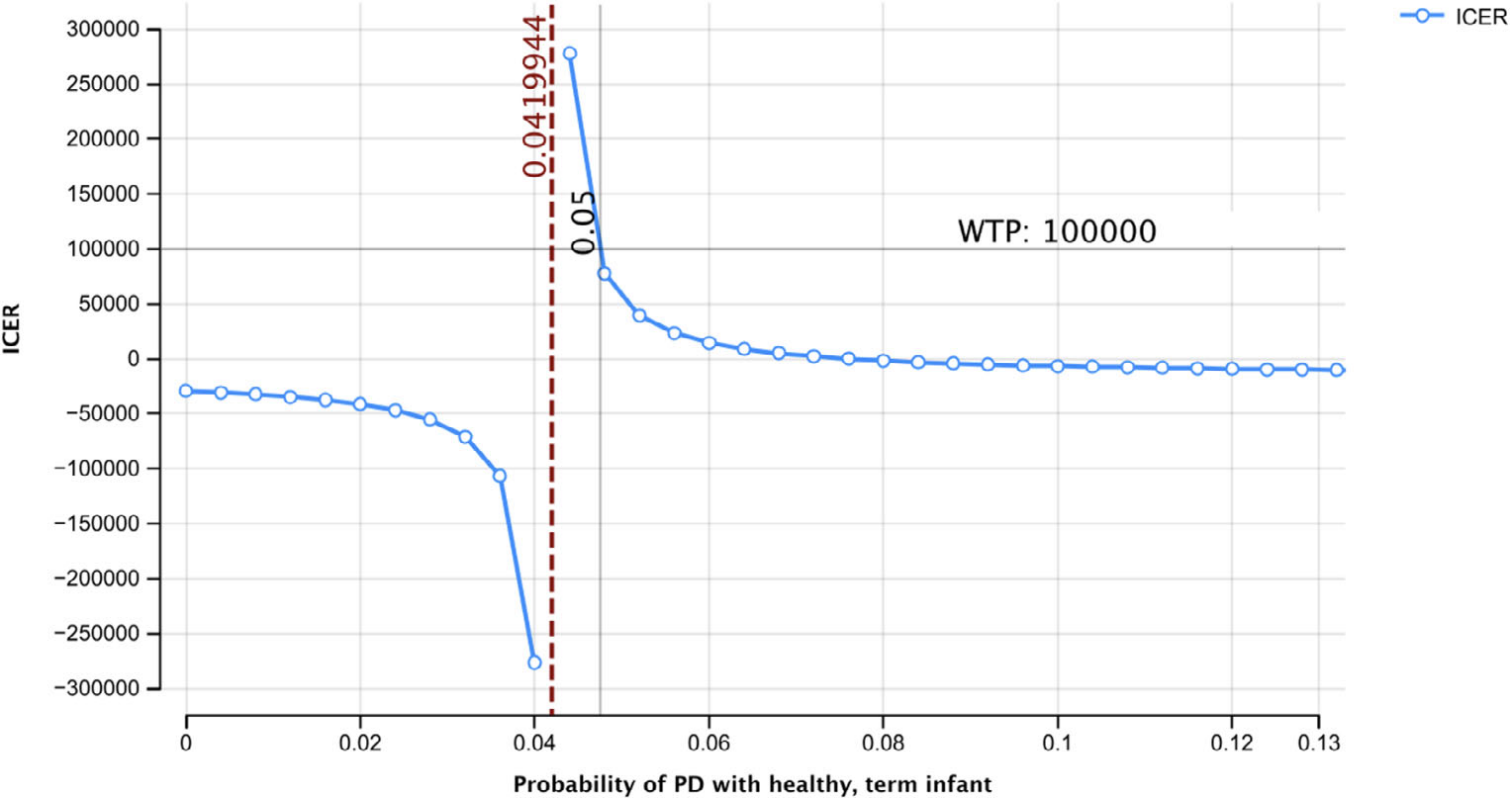
One‐way sensitivity analysis of the probability of developing perinatal depression with a healthy, term infant without treatment. MBCT is only cost‐effective when this probability remains above 4.2%. The baseline probability used in the model was 13.2%. WTP, willingness‐to‐pay.

Another driver of the model was the rate of preterm birth for those dyads in which the mother received MBCT treatment. The inputs for preterm birth rate for those who did not receive MBCT treatment versus those who did were 10.49% and 9.6%, respectively. The treatment remained cost‐saving until the preterm birth rate for patients given the MBCT treatment reached 10.26%, at which point it became cost‐effective (Figure 3). Additionally, the model was only dominant when the probability of developing perinatal depression after a healthy, full‐term delivery without receiving MBCT treatment was above 4.2% (Figure 4).

**FIGURE 5:**
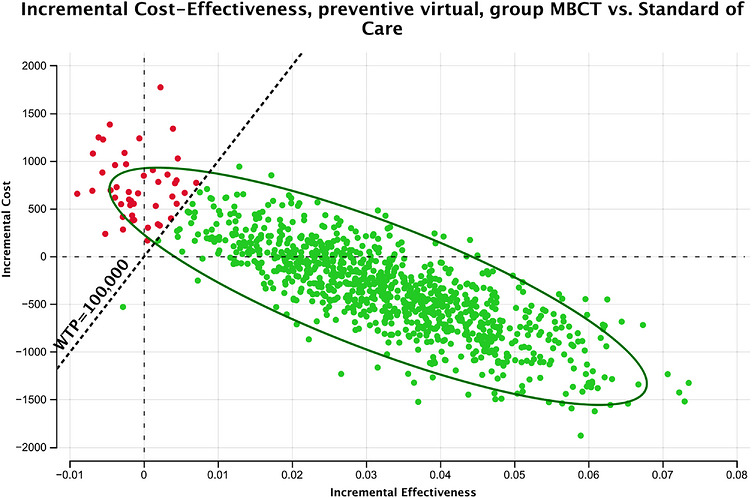
Monte Carlo simulation. Green dots represent trials in which Mindfulness‐Based Cognitive Therapy (MBCT) care was cost‐effective and red dots represent trials in which it was not. A total of 96.67% of the simulated 10,000 trials resulted in MBCT treatment being cost‐effective. MBCT, Mindfulness‐Based Cognitive Therapy; WTP, willingness‐to‐pay.

To address the uncertainty of the model inputs simultaneously, we used a Monte Carlo analysis to simulate 10,000 trials (Figure 5). Virtual group MBCT was found to be the cost‐effective method in 96.7% of trials. Each point in the figure represents one of 10,000 random trials. The green points represent trials where MBCT treatment was cost‐effective, while red points represent care as usual being the cost‐effective route. Each trial uses a different value of each variable selected by random chance. The ellipse represents the 95% confidence interval. The willingness‐to‐pay threshold is represented by the dashed line under which 96.7% of the trials occurred.

## 4 DISCUSSION

We found that virtual, group, SW‐led MBCT resulted in better outcomes and was cost‐saving. Compared to care as usual, which does not reliably include preventive mental health care on screened patients determined to be high‐risk, MBCT led to a reduction in cases of preterm deliveries, perinatal depression and psychosis, maternal suicides, neurodevelopmental delay, and neonatal demise. While mental health support screening and intervention are currently available, they are not universally implemented for high‐risk patients. In fact, less than 15% of eligible patients may have access to in‐person counseling [64]. Given that some of the lag in uptake of high‐risk interventions may be due to associated costs of these programs, we hope to show through this model that SW‐led, virtual, group MBCT is cost‐effective and worth implementing universally. When only costs to the hospital were included, virtual group MBCT remained cost‐saving, suggesting that it would be advantageous to insurance companies to cover the cost of virtual, group, SW‐led MBCT preventive care during pregnancy.

A key factor that makes preventive virtual, group MBCT cost‐saving is the relatively low cost of facilitating the care sessions compared to treating the potential negative outcomes from a societal standpoint. Two of the biggest costs in this model were associated with lifetime costs of treating a neurodevelopmental disorder as well as the lost wages resulting from maternal suicide. Cost of lifetime treatment of cerebral palsy was used to represent the cost of neurodevelopmental disorder. Rates of both outcomes were lower using MBCT preventive care given its impact on preterm birth and perinatal depression rates. To address whether MBCT would still be cost‐saving without these high costs, a sensitivity analysis was conducted setting the cost of maternal death from suicide as well as lifetime costs of a neurodevelopmental disorder to $0. This version of the model reflected hospital‐specific costs rather than the societal costs of the outcomes. Even with both large costs removed, the MBCT preventive care plan was cost‐saving.

Preventive MBCT is cost‐saving until intervention costs exceed $800 and then cost‐effective until $4014. The predicted cost for providing virtual, group, SW‐led MBCT prevention care was $480 per person, below the cost‐saving threshold [19]. This low cost is due in part to the use of SWs to facilitate the MBCT sessions, rather than psychologists, and holding the sessions online and in groups. Virtual mental health care has shown equally high patient satisfaction rates to in‐person mental health care in addition to improved wait times and cost‐savings [22]. Recent pilot randomized controlled trials are similarly showing that virtual modalities for perinatal mental health care show high recruitment, adherence, and satisfaction rates [65, 66]. Group therapy approaches have similarly been shown to be efficacious for treating depression [67]. A critical element of these counseling sessions is providing high‐risk patients with the tools to independently deal with potential stressors, which can be effectively executed by SWs [19]. Peer support aspects of perinatal group‐based care models have also been associated with improved maternal/child outcomes [23, 24]. Furthermore, SWs can be effectively trained in MBCT care in just 6 hours over two sessions [19], and virtual care can be delivered via established health‐encrypted virtual meeting software.

One‐way sensitivity analyses revealed that the probability of preterm delivery after MBCT treatment and the probability of developing perinatal depression without treatment were two key drivers of the model (Figures 3 and 4). For the virtual, group, SW‐led MBCT model to remain dominant, the probability of a preterm delivery following the intervention had to be below 10.26%, above which the strategy became only cost‐effective. The prevalence of preterm delivery is 10.49% without MBCT treatment [35], indicating that if MBCT only reduces preterm birth rates by 0.23%, it is still cost‐saving. Furthermore, if MBCT treatment has no impact on preterm birth rates, it is still a cost‐effective strategy over standard care. Similarly, MBCT was no longer cost‐effective when the perinatal depression rate was lower than or equal to 4.2%, as a low probability of developing perinatal depression would place the associated costs of preventive MBCT above the $100,000 per gained QALY cost‐effectiveness threshold. As current domestic rates of perinatal depression are approximately 14% [2], it is unlikely that rates would reduce this much in coming years, particularly in high‐risk populations.

Given the nature of this study, we were limited by the existing literature on the conditions included and the efficacy of care models. Data on perinatal psychosis rates and their impact on other outcomes were lacking. Unlike perinatal depression rates, it was assumed that MBCT would have no impact on development of perinatal psychosis. Additionally, though there is initial evidence showing the efficacy of preventive MBCT in a virtual, group format, there are limited studies using this intervention in the literature and not all of the addressed outcomes were measured variables within them [17, 19]. To approximate the impact of this counseling intervention on relevant outcomes beyond reduced depression rates, other studies utilizing similar mental health care approaches were employed. Existing meta‐analyses revealing the similar impacts of both MBIs and CBTs were referenced to account for these substitutions [34]. This model did not include additional adverse outcomes associated with the development of perinatal depression beyond maternal suicide. Perinatal depression is also associated with a lack of motivation and perceived self‐efficacy, leading to reduced productivity and social functioning [68, 69]. However, these potential societal costs of absenteeism and/or presenteeism were not included in the model, though recent estimates suggest related societal costs may be substantial [10]. Lastly, this health economic model is entirely theoretical based on existing literature. Therefore, limitations in the literature, such as potential unrecorded issues with intervention adherence and uptake, may result in possible overestimation of effect sizes in the model. It is possible that the predicted cost savings and prevented negative health outcomes from implementing universal virtual, group, SW‐led MBCT may not match real‐life outcomes; however, sensitivity analyses conducted for model drivers within their ranges of uncertainty as well as the Monte Carlo simulation predict that overly large variability with model results are unlikely.

## 5 CONCLUSION

Perinatal depression is a leading cause of preventable maternal mortality. Rates of perinatal depression were estimated to be 13.2% in 2018 but as high as 33% during the COVID‐19 pandemic [1, 70]. Our model provides evidence that referral to preventive MBCT for pregnancies at high risk of perinatal depression yields better outcomes than the current standard of care, with low associated costs by utilizing a virtual, group setting led by social workers. Even when the cost of maternal death and lifetime treatment of a neurodevelopmental disorder were removed from the model, costs shouldered by individuals rather than insurance companies or hospitals, preventive MBCT was still a cost‐saving strategy relative to the standard of care. The cost‐saving nature of preventive MBCT makes it a strategy that insurance companies should consider for coverage and that perinatal health systems should consider trialing.

## CONFLICT OF INTEREST STATEMENT

The authors declare no conflicts of interest.

## FUNDING INFORMATION

The authors received no specific funding for this work.

## Data Availability

Data sharing is not applicable to this article as no datasets were generated or analyzed during the current study.
